# Prediction of psychosis and functional outcome in children and adolescents at clinical high-risk of psychosis

**DOI:** 10.1192/j.eurpsy.2026.10374

**Published:** 2026-07-31

**Authors:** F. Schultze-Lutter, C. Michel, BEARS-Kid consortium

**Affiliations:** 1 Department of Psychiatry and Psychotherapy, Heinrich-Heine University, Düsseldorf, Germany; 2 University Hospital of Child and Adolescent Psychiatry and Psychotherapy, University of Bern, Bern, Switzerland

## Abstract

**Introduction:**

Psychoses cause great burden, already in children and adolescents, and early-onset psychoses show some distinct features compared to adult-onset psychoses. Furthermore, clinical high risk (CHR) criteria, which had been developed and validated predominately in adult samples, were associated with significantly lower conversion rates in children and young adolescents, and preventive interventions had lesser effect in this age group. This indicates that children and adolescents might make special demands on the early detection and treatment of psychosis.

**Objectives:**

To examine how well current CHR criteria predict psychosis in underage outpatients over the course of two years.

**Methods:**

One-hundred seventy-six 8- to 17-year-olds with any CHR criteria according to the ultra-high risk (UHR) and basic symptom concept as well as 306 inpatient and 233 community controls were recruited as part of the multicenter naturalistic Bi-national Evaluation of At-Risk Symptoms in children and adolescents (BEARS-Kid) study and naturalistically followed-up annually over two years.

**Results:**

Altogether 10 conversions to psychosis occurred during the follow-up, eight within the first and two within the second year past baseline. Thus, the conversion rate was 5.7% in relation to the number of baseline interviews and was 8.5% and 14.5% in relation to the 1- and 2-year follow-up samples. Six converters had been 16-17 years old at baseline, three 14-15 years and one had turned 12 years within the time of the baseline assessment; 70% were male. At baseline, n=6 had met both ultra-high risk (UHR) and basic symptom criteria, and n=2 each only UHR and only basic symptom criteria. One converter each had initially been assessed as part of a community control and an inpatient control sample.

**Conclusion:**

In line with earlier reports, our findings confirm that the psychosis-predictive value of current CHR criteria is age-dependent but only slightly lower in children and adolescent.

**Disclosure of Interest:**

None Declared

